# Liposomal delivery and polyethylene glycol-liposomal oxaliplatin for the treatment of colorectal cancer (Review)

**DOI:** 10.3892/br.2014.249

**Published:** 2014-03-12

**Authors:** CHUANG YANG, ZHONG-XUE FU

**Affiliations:** 1 Department of General Surgery, Third People’s Hospital of Mianyang, Mianyang, Sichuan 621000, P.R. China; 2 Department of Gastrointestinal Surgery, The First Affiliated Hospital, Chongqing Medical University, Chongqing, Chongqing 400016, P.R. China

**Keywords:** colorectal cancer, oxaliplatin, drug delivery system, liposomes

## Abstract

Oxaliplatin is effective for the treatment of advanced colorectal cancer; however, its application is restricted due to its dose-limiting toxicity. Liposomes are sphere-shaped vesicles consisting of one or more phospholipid bilayers. Liposomes as drug carriers are characterized by delayed release, lesion targeting and may be used as a drug-delivery system to decrease the side effects of cytotoxic drugs. Active targeting modification of liposomes may change the biological distribution of the anticancer agents, reduce or reverse multidrug resistance of tumor cells and enhance the effects of anticancer therapy. Based on the characteristics mentioned above, the aim of the present review was to demonstrate that polyethylene glycol-liposomes containing oxaliplatin may offer advantages for the treatment of colorectal cancer in clinical practice.

## 1. Introduction

Colorectal cancer (CRC) is currently the third most common malignancy worldwide. Radical resection is curative for only ~50% of the patients (1), whereas for the majority of patients with advanced-stage or metastatic disease, or for those who cannot be treated with radical resection, chemotherapy is the main treatment of choice (2,3). The survival rate of patients with metastatic CRC has significantly improved with the application of molecularly-targeted drugs, such as oxaliplatin.

Oxaliplatin, a diaminocyclohexane platinum compound, interrupts the replication and transcription of DNA (4). Oxaliplatin is the third generation of platinum drugs after cisplatin and carboplatin and is effective in the treatment of CRC, particularly CRC that is resistant to 5-fluorouracil (5,6). Oxaliplatin may also be effective for the treatment of tumors that do not respond adequately to cisplatin and carboplatin, as well as drug-resistant tumors. Oxaliplatin acts synergistically with other anticancer drugs, such as fluorouracil, topoisomerase inhibitors and microtubule inhibitors (7,8).

Satisfactory clinical results have also been achieved with the combined application of oxaliplatin and molecular-targeted drugs, such as bevacizumab and cetuximab, administered intravascularly, with a median survival time of 30 months in the majority of the patients and of >3 years in certain patients (9). Although a number of studies indicated that the combined application of oxaliplatin with other chemotherapeutics and molecular-targeted drugs may achieve good clinical results in the treatment of CRC, the associated toxicity and side effects, such as neurotoxicity, cardiotoxicity, gastrointestinal reactions, hemorrhage and hypersensitivity, may outweigh the benefits of the treatment (10–14).

The nature of the active species generated *in vivo*, uptake, efflux, intracellular trafficking or insufficient diffusion in tumor tissues, resulting in decreased curative effects and increased toxicity for certain chemotherapeutic agents (15). Oxaliplatin therapy based on a simple vesicular delivery system may reduce the potential side effects, target specific organs and improve the therapeutic effects.

## 2. Liposomes as anticancer drug carriers

Over the last few decades, liposomes have been widely accepted as agent nanocarriers. Liposomes are small, spherical artificial vesicles that consist of cholesterol and natural non-toxic phospholipids. Due to their size, biocompatibility and hydrophobic and hydrophilic properties, liposomes are promising drug delivery systems. Liposomes have a phospholipid bilayer structure that is compatible with cell membranes (16); therefore, they are among the most effective drug carriers into cells, with slow-releasing and targeting characteristics and the ability to reduce side effects (17,18). Drugs coated in liposomes are slowly released through infiltration or degradation of liposomes, leading to a reduction in the metabolism and excretion of drugs by the body and prolonged time of action. Liposomes as exogenous substances may be devoured by macrophages; however, liposomal drugs administered intravenously may selectively act on the mononuclear macrophage system (19,20). The drugs delivered by surface-modified liposomes escape being taken up by the endodermis system, act specifically on target organs, increase drug concentration in these organs and improve the therapeutic effects, while reducing toxicity (21,22). In addition, drugs insulated by bilayer liposomes are stable; therefore, surface-modified liposomes exhibit advantages in the treatment of a number of diseases, particularly cancer.

The toxicity of drugs coated by ordinary liposomes may be reduced; however, the therapeutic effects are severely affected as the drugs lose their bioactivity. Previous studies demonstrated that different types of liposomes may be obtained based on liposome modifiers (23,24) and modified liposomes may be more effective drug delivery systems.

Liposomes are broadly divided into the following 3 groups according to their different properties:

### Long-circulating liposomes (stealth liposomes)

The surface conformation of the phospholipid bilayer structure is modified by adding gangliosides or a polyethylene glycol (PEG) derivative possessing a flexible chain that occupies the space immediately adjacent to the liposome surface, tends to exlcude other macromolecules from this space (25,26), and prevent blood plasma opsonins binding to the liposome surface. Consequently, PEG decreases the recognition of liposomes by the mononuclear phagocyte system and enables liposomes to remain stable in the circulation and exhibit a prolonged half-life (27,28). This type of liposome has been applied in clinical practice and achieved satisfactory effects in individualized treatment, such as treatment for hepatocellular carcinoma with doxorubicin liposomes and ovarian carcinoma with paclitaxel liposomes (29–31).

### Active targeting liposomes

Liposomes targeting antibodies, peptides, glycoside residues, hormones and receptors. The ligands are constructed on the phospholipid bilayer structure (32–37); thus, the liposomes are able to identify and migrate to the target organ and release the anticancer agent.

### Liposomes with special properties

This type of liposomes includes pH-sensitive, thermosensitive, magnetic and positive liposomes (38–41). There are several types of liposomes; however, there are currently no uniform standards regarding their application and these liposomes should be selected according to the different treatment or experimental requirements.

## 3. PEG-liposomes with enhanced permeability and retention (EPR) effect

It is crucial to investigate PEG-liposomes with EPR effect, as the EPR effect of tumors on macromolecules is a common phenomenon. Previous studies reported that new vessel formation is the basis of solid tumor growth (42,43). Compared to normal tissues, capillaries in tumor tissues exhibit the following characteristics: irregular wall structure, dilated lumen, defective wall and loosely arranged endothelial cells (44), incomplete lymphangiogenesis and defective lymphatic return. Therefore, these abnormalities may result in the penetration of macromolecules and lipid granules from the lumen into the surrounding tissues, which is referred to as the EPR of solid tumor tissues. The pathological characteristics of solid tumors may enable the macromolecular anticancer drugs to achieve a highly distributed concentration in tumor tissues (45,46).

Currently available evidence indicates that liposomes accumulate in solid tumor tissues and efficiently inhibit tumor growth (47,48), which is associated with the EPR effect. Due to the increased permeability of the solid tumor vessels to macromolecules and the incomplete lymphatic clearance, the lipid granules may remain in the tumor tissues for weeks or even months (49). Long-circulating liposomes, immune liposomes and liposomes with special properties may increase the drug cumulative effect in tumor tissues due to their active organ targeting (50,51).

## 4. PEG-liposomal oxaliplatin for the treatment of CRC

**Figure 1:**
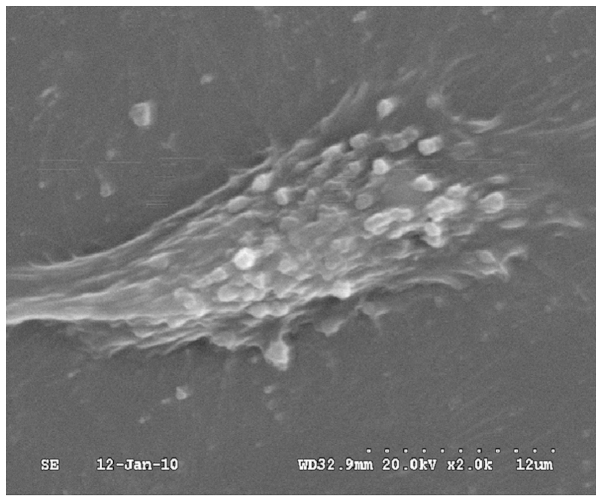
Scanning electron microscopy reveals polyethylene glycol (PEG)-liposome coherence to cells. The PEG-liposomes were incubated with SW480 cells at 4°C to allow binding (30 min). The unbound PEG-liposomes were removed by extensively washing the cells with ice-cold phosphate-buffered saline.

**Figure 2:**
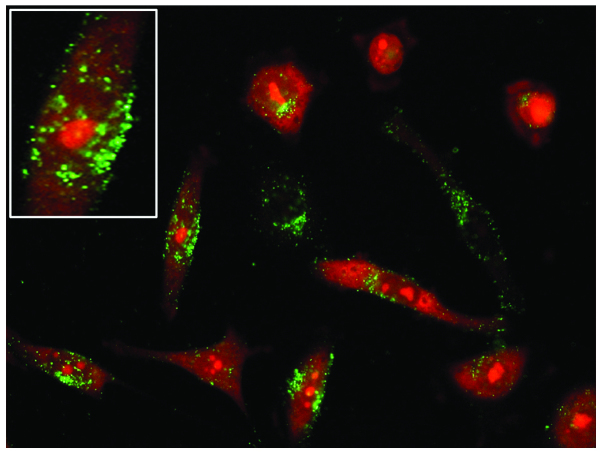
Cell internalizaton of polyethylene glycol (PEG)-liposomes. The PEG-liposomes conjugated with SW480 cells after 2 h. The cells were incubated with propidium iodide, which stained the nuclei red and DiO-labelled PEG-liposomes, which were stained green. A significant number of PEG-liposomes is aggregated within the cells (magnification, ×400).

Regular liposomes have a low encapsulation efficiency and poor stability. Long-circulating liposomes modified by PEG are more stable in the plasma and have a longer circulation time and relatively lower toxicity (52). Moreover, our previous *in vitro* study demonstrated the easy coherence of PEG-liposomes to cells (Fig. 1), their internalization and their subsequent intracellular route (Fig. 2). However, it is the size of the particles that determines the entry pathway (53).

As oxaliplatin has a different antineoplastic spectrum and no cross-resistance with cisplatin, it exerts a good curative effect on advanced CRC. Liposome studies on oxaliplatin and its derivatives are attracting increasing attention, particularly regarding liposomes modified by PEG. The surface modification of PEG-liposomes with specific ligands, such as monoclonal antibodies, peptides, folic acid and transferrin, may further improve the active targeting efficiency of liposomes (25,30,54).

Considering the water solubility of oxaliplatin, the low encapsulation efficiency of liposomes is the main concern. A previous study reported that the encapsulation efficiency of oxaliplatin liposomes was ~30% (55), whereas PEG-liposomal oxaliplatin prepared with the film dispersion method by Zalba *et al* (56) exhibited an encapsulation efficiency of ≤35%. Liposomes prepared by optimizing the preparation technique, as described by Liu *et al* (57), exhibited an encapsulation efficiency of ≤69.1%. Our previous study demonstrated that the encapsulation efficiency of PEG-liposomal oxaliplatin was ~58% (58). These differences in the encapsulation efficiency may be associated with the different preparation techniques.

The action time of oxaliplatin coated with liposomes was significantly prolonged and its toxicity against normal cells was significantly reduced. High concentrations of oxaliplatin were obtained in the cytoplasm and then combined with nuclear DNA as >95% of PEG-liposomal oxaliplatin was internalized by CRC cells (59). Treatments for CRC with PEG-liposomal oxaliplatin are currently at the research phase. Doi *et al* (60) investigated the therapeutic effect of PEG-liposomal oxaliplatin in a mouse CRC model and demonstrated that PEG-liposomal oxaliplatin exerted a significant inhibitory effect on tumors compared to free oxaliplatin (>50%), with an increased drug content in tumors. Jain *et al* (61) coated oxaliplatin with hyaluronic acid-chitosan, administered the drug to nude mice bearing TH29 colorectal tumor xenografts and found that the drug concentration in the tumor tissues reached a peak value 24 h after administration. Radioisotope scanning revealed that the liposomes had accumulated in the colorectal tumor 24 h after administration.

Abu Lila *et al* (62) recently reported a higher cumulative distribution effect of PEG-liposomal oxaliplatin in colorectal tumor tissues through a comparative study of CRC, lung cancer and melanoma. Different types of tumor cells can take up different amounts of drug-carrying liposomes, indicating that the permeability of different tumor vessels is a factor affecting tumor localization and the antitumor effects of drug-carrying liposomes (63). In our previous experiment, oxaliplatin was coated with DSPE-PEG2000-modified liposomes and the PEG-liposomes exerted a significant antitumor effect *in vivo* and *in vitro* (51,64). Further investigations revealed that Fas/Fas ligand and the caspase pathway may be involved in the apoptosis-inducing effects of PEG-liposomal oxaliplatin on CRC cells (65).

Tumors are unable to grow without vessels and capillaries are the foundation of tumor survival. Taking advantage of the properties of PEG-liposomes may allow drugs to migrate to the target organ by constructing a vascular-targeting substance, such as vascular endothelial growth factor (VEGF) and VEGF monoclonal antibody peptides, on the surface of liposomes (66). Therefore, the preparation of PEG-liposomal oxaliplatin is of great clinical significance.

## 5. Conclusion

Oxaliplatin exerts a good curative effect on CRC, fully embodying the advantages of platinum drugs. However, there is a need to reduce the toxic side effects of oxaliplatin. As a novel type of drug carrier, liposomes exhibit good targeting properties, slow-releasing potential, high stability and low toxicity following surface modification. The active targeting modifications are significant for altering the biological distribution of antitumor agents, reducing or reversing the multidrug resistance of tumor cells and improving the efficiency of anticancer drugs. Further studies investigating the effects of PEG-liposomal oxaliplatin on CRC are required to establish the advantages of its application in clinical practice.

## References

[1] Spolverato G, Ejaz A, Azad N, Pawlik TM (2013). Surgery for colorectal liver metastases: The evolution of determining prognosis. World J Gastrointest Oncol.

[2] Alberts SR, Sargent DJ, Nair S (2012). Effect of oxaliplatin, fluorouracil, and leucovorin with or without cetuximab on survival among patients with resected stage III colon cancer: a randomized trial. JAMA.

[3] Garcia-Foncillas J, Diaz-Rubio E (2010). Progress in metastatic colorectal cancer: growing role of cetuximab to optimize clinical outcome. Clin Transl Oncol.

[4] Wiseman LR, Adkins JC, Plosker GL (1999). Oxaliplatin: a review of its use in the management of metastatic colorectal cancer. Drugs Aging.

[5] Simpson D, Dunn C, Curran M, Goa KL (2003). Oxaliplatin: a review of its use in combination therapy for advanced metastatic colorectal cancer. Drugs.

[6] Yang DY, Li Y, Liu JH (2013). Efficacy and tolerance of maintenance therapy in patients with incurable advanced colorectal cancer. J Southern Med Uni.

[7] Brodowicz T, Ciuleanu TE, Radosavljevic D (2013). FOLFOX4 plus cetuximab administered weekly or every second week in the first-line treatment of patients with KRAS wild-type metastatic colorectal cancer: a randomized phase II CECOG study. Ann Oncol.

[8] Douillard JY, Oliner KS, Siena S (2013). Panitumumab-FOLFOX4 treatment and RAS mutations in colorectal cancer. N Engl J Med.

[9] Messersmith WA, Jimeno A, Jacene H (2010). Phase I trial of oxaliplatin, infusional 5-fluorouracil, and leucovorin (FOLFOX4) with erlotinib and bevacizumab in colorectal cancer. Clin Colorectal Cancer.

[10] McWhinney SR, Goldberg RM, McLeod HL (2009). Platinum neurotoxicity pharmacogenetics. Mol Cancer Ther.

[11] Ochenduszko SL, Krzemieniecki K (2010). Targeted therapy in advanced colorectal cancer: more data, more questions. Anticancer Drugs.

[12] Cortejoso L, Garcia MI, Garcia-Alfonso P (2013). Differential toxicity biomarkers for irinotecan- and oxaliplatin-containing chemotherapy in colorectal cancer. Cancer Chemother Pharmacol.

[13] Di Francia R, Siesto RS, Valente D (2012). Pharmacogenomics panel test for prevention toxicity in patient who receive fluoropirimidine/oxaliplatin-based therapy. Eur Rev Med Pharmacol Sci.

[14] Hoff PM, Saad ED, Costa F (2012). Literature review and practical aspects on the management of oxaliplatin-associated toxicity. Clin Colorectal Cancer.

[15] Olszewski U, Hamilton G (2010). A better platinum-based anticancer drug yet to come?. Anticancer Agents Med Chem.

[16] Patil YP, Jadhav S (2014). Novel methods for liposome preparation. Chem Phys Lipids.

[17] Jain RL, Shastri JP (2011). Study of ocular drug delivery system using drug-loaded liposomes. Int J Pharm Investig.

[18] Suntres ZE (2011). Liposomal antioxidants for protection against oxidant-induced damage. J Toxicol.

[19] Pagano RE, Weinstein JN (1978). Interactions of liposomes with mammalian cells. Annu Rev Biophys Bioeng.

[20] Yefimova SL, Kurilchenko IY, Tkacheva TN (2012). Comparative study of dye-loaded liposome accumulation in sensitive and resistant human breast cancer cells. Exp Oncol.

[21] Saffari M, Shirazi HF, Oghabian MA (2013). Preparation and in-vitro evaluation of an antisense-containing cationic liposome against non-small cell lung cancer: a comparative preparation study. Iran J Pharm Res.

[22] Preiss MR, Bothun GD (2011). Stimuli-responsive liposome-nanoparticle assemblies. Expert Opin Drug Deliv.

[23] Rangger C, Helbok A, Sosabowski J (2013). Tumor targeting and imaging with dual-peptide conjugated multifunctional liposomal nanoparticles. Int J Nanomedicine.

[24] Li X, Zhang J, Wang DK (2013). Anti-tumor activity of folate receptor targeting docetaxel-loaded membrane-modified liposomes. Acta Pharma Sinica.

[25] Nag OK, Awasthi V (2013). Surface engineering of liposomes for stealth behavior. Pharmaceutics.

[26] Noble GT, Stefanick JF, Ashley JD (2014). Ligand-targeted liposome design: challenges and fundamental considerations. Trends Biotechnol.

[27] Immordino ML, Dosio F, Cattel L (2006). Stealth liposomes: review of the basic science, rationale, and clinical applications, existing and potential. Int J Nanomedicine.

[28] Akbarzadeh A, Rezaei-Sadabady R, Davaran S (2013). Liposome: classification, preparation, and applications. Nanoscale Res Lett.

[29] Allen TM, Cullis PR (2013). Liposomal drug delivery systems: from concept to clinical applications. Adv Drug Deliv Rev.

[30] Samad A, Sultana Y, Aqil M (2007). Liposomal drug delivery systems: an update review. Curr Drug Deliv.

[31] Cattel L, Ceruti M, Dosio F (2003). From conventional to stealth liposomes: a new frontier in cancer chemotherapy. Tumori.

[32] Smith-Jones PM, Vallabhajosula S, Navarro V (2003). Radiolabeled monoclonal antibodies specific to the extracellular domain of prostate-specific membrane antigen: preclinical studies in nude mice bearing LNCaP human prostate tumor. J Nucl Med.

[33] Yan Z, Zhan C, Wen Z (2011). LyP-1-conjugated doxorubicin-loaded liposomes suppress lymphatic metastasis by inhibiting lymph node metastases and destroying tumor lymphatics. Nanotechnology.

[34] Brignole C, Marimpietri D, Gambini C (2003). Development of Fab’ fragments of anti-GD(2) immunoliposomes entrapping doxorubicin for experimental therapy of human neuroblastoma. Cancer Lett.

[35] Yang Y, Yan Z, Wei D (2013). Tumor-penetrating peptide functionalization enhances the anti-glioblastoma effect of doxorubicin liposomes. Nanotechnology.

[36] Yan Z, Wang F, Wen Z (2012). LyP-1-conjugated PEGylated liposomes: a carrier system for targeted therapy of lymphatic metastatic tumor. J Control Release.

[37] Ishida O, Maruyama K (1998). Transferrin conjugated PEG-liposomes as intracellular targeting carrier for tumor therapy. Jpn J Clin Med.

[38] Rane S, Prabhakar B (2013). Optimization of paclitaxel containing pH-sensitive liposomes by 3 factor, 3 level box-behnken design. Indian J Pharm Sci.

[39] Dicheva BM, Koning GA (2014). Targeted thermosensitive liposomes: an attractive novel approach for increased drug delivery to solid tumors. Expert Opin Drug Deliv.

[40] Linemann T, Thomsen LB, Jardin KG (2013). Development of a novel lipophilic, magnetic nanoparticle for in vivo drug delivery. Pharmaceutics.

[41] Alinaghi A, Rouini MR, Johari Daha F (2014). The influence of lipid composition and surface charge on biodistribution of intact liposomes releasing from hydrogel-embedded vesicles. Int J Pharm.

[42] Iversen PO (2003). Angiogenesis and hematological malignancies. J Norw Med Assoc.

[43] Bisacchi D, Benelli R, Vanzetto C (2003). Anti-angiogenesis and angioprevention: mechanisms, problems and perspectives. Cancer Detect Prev.

[44] Abdollahi A, Folkman J (2010). Evading tumor evasion: current concepts and perspectives of anti-angiogenic cancer therapy. Drug Resist Updat.

[45] Waite CL, Roth CM (2012). Nanoscale drug delivery systems for enhanced drug penetration into solid tumors: current progress and opportunities. Crit Rev Biomed Eng.

[46] Prabhakar U, Maeda H, Jain RK (2013). Challenges and key considerations of the enhanced permeability and retention effect for nanomedicine drug delivery in oncology. Cancer Res.

[47] Taurin S, Nehoff H, Greish K (2012). Anticancer nanomedicine and tumor vascular permeability; Where is the missing link?. J Control Release.

[48] Greish K (2010). Enhanced permeability and retention (EPR) effect for anticancer nanomedicine drug targeting. Methods Mol Biol.

[49] Maeda H, Bharate GY, Daruwalla J (2009). Polymeric drugs for efficient tumor-targeted drug delivery based on EPR-effect. Eur J Pharm Biopharm.

[50] Karn PR, Cho W, Hwang SJ (2013). Liposomal drug products and recent advances in the synthesis of supercritical fluid-mediated liposomes. Nanomedicine (Lond).

[51] Yang C, Liu HZ, Lu WD (2011). PEG-liposomal oxaliplatin potentialization of antitumor efficiency in a nude mouse tumor-xenograft model of colorectal carcinoma. Oncol Rep.

[52] Nakamura H, Doi Y, Abu Lila AS (2013). Sequential treatment of oxaliplatin-containing PEGylated liposome together with S-1 improves intratumor distribution of subsequent doses of oxaliplatin-containing PEGylated liposome. Eur J Pharm Biopharm.

[53] Rejman J, Oberle V, Zuhorn IS, Hoekstra D (2004). Size-dependent internalization of particles via the pathways of clathrin- and caveolae-mediated endocytosis. Biochem J.

[54] Hood RR, Shao C, Omiatek DM (2013). Microfluidic synthesis of PEG- and folate-conjugated liposomes for one-step formation of targeted stealth nanocarriers. Pharm Res.

[55] Abu Lila AS, Doi Y, Nakamura K (2010). Sequential administration with oxaliplatin-containing PEG-coated cationic liposomes promotes a significant delivery of subsequent dose into murine solid tumor. J Control Release.

[56] Zalba S, Navarro I, Troconiz IF (2012). Application of different methods to formulate PEG-liposomes of oxaliplatin: evaluation in vitro and in vivo. Eur J Pharm Biopharm.

[57] Liu XP, Geng DQ, Xu HX (2008). Research on the preparation of oxaliplatin liposome. J Wuhan Univ Technol.

[58] Yang C, Liu HZ, Fu ZX, Lu WD (2011). Oxaliplatin long-circulating liposomes improved therapeutic index of colorectal carcinoma. BMC Biotechnology.

[59] Tippayamontri T, Kotb R, Paquette B (2011). Cellular uptake and cytoplasm/DNA distribution of cisplatin and oxaliplatin and their liposomal formulation in human colorectal cancer cell HCT116. Invest New Drugs.

[60] Doi Y, Okada T, Matsumoto H (2010). Combination therapy of metronomic S-1 dosing with oxaliplatin-containing polyethylene glycol-coated liposome improves antitumor activity in a murine colorectal tumor model. Cancer Sci.

[61] Jain A, Jain SK, Ganesh N (2010). Design and development of ligand-appended polysaccharidic nanoparticles for the delivery of oxaliplatin in colorectal cancer. Nanomedicine.

[62] Abu Lila AS, Matsumoto H, Doi Y (2012). Tumor-type-dependent vascular permeability constitutes a potential impediment to the therapeutic efficacy of liposomal oxaliplatin. Eur J Pharm Biopharm.

[63] Abu Lila AS, Ichihara M, Shimizu T (2013). Ex-vivo/in-vitro anti-polyethylene glycol (PEG) immunoglobulin M production from murine splenic B cells stimulated by PEGylated liposome. Biol Pharm Bull.

[64] Yang C, Liu HZ, Fu ZX (2012). Effects of PEG-liposomal oxaliplatin on apoptosis, and expression of Cyclin A and Cyclin D1 in colorectal cancer cells. Oncol Rep.

[65] Yang C, Liu HZ, Fu ZX (2012). PEG-liposomal oxaliplatin induces apoptosis in human colorectal cancer cells via Fas/FasL and caspase-8. Cell Biol Int.

[66] Wicki A, Rochlitz C, Orleth A (2012). Targeting tumor-associated endothelial cells: anti-VEGFR2 immunoliposomes mediate tumor vessel disruption and inhibit tumor growth. Clin Cancer Res.

